# One-year survival of aneurysmal subarachnoid hemorrhage after airplane transatlantic transfer – a monocenter retrospective study

**DOI:** 10.1186/s12871-024-02532-7

**Published:** 2024-04-12

**Authors:** Frédéric Martino, Antoine Fleuri, Nicolas Engrand, Amélie Rolle, Michel Piotin, Michel Carles, Delphine Delta, Laurent Do, Adrien Pons, Patrick Portecop, Mathys Sitcharn, Marc Valette, Laurent Camous, Jean-David Pommier, Alexandre Demoule

**Affiliations:** 1 Réanimation Médicale et Chirurgicale, CHU de la Guadeloupe, Route de Chauvel, Pointe à Pitre Cedex, Guadeloupe, 97159 France; 2 Université Paris Cité and Université des Antilles, INSERM, Biologie intégrée du globule rouge, Paris, France; 3 Service d’Accueil des Urgences, CHU de la Guadeloupe, Pointe à Pitre, Guadeloupe, France; 4 Neuro-Intensive Care Unit – Anesthesiology, Rothschild Foundation Hospital, Paris, France; 5 Anesthésie et Médecine Péri Opératoire, CHU de la Guadeloupe, Pointe à Pitre, Guadeloupe, France; 6 Département de Neuroradiologie Interventionnelle, Hôpital de la Fondation Rothschild, Paris, France; 7 Service de Maladies Infectieuses et Tropicales, CHU de Nice, Nice, France; 8 Université Cote d’Azur, INSERM, UMRU1065 Centre Méditerranéen de Médecine Moléculaire, Nice, France; 9 Service de Neurochirurgie, CHU de la Guadeloupe, Pointe à Pitre, Guadeloupe, France; 10 SAMU- SMUR, CHU de la Guadeloupe, Pointe à Pitre, Guadeloupe, France; 11 Service de Médecine Intensive - Réanimation (Département R3S), AP-HP, Groupe Hospitalier Universitaire APHP-Sorbonne Université, site Pitié-Salpêtrière, Paris, France; 12 Sorbonne Université, INSERM, UMRS1158 Neurophysiologie Respiratoire Expérimentale et Clinique, Paris, France

**Keywords:** Aneurysmal subarachnoid hemorrhage, Airplane transfer, One-year mortality, Safety, Mechanical ventilation

## Abstract

**Background:**

Aneurysmal subarachnoid hemorrhage (aSAH) is preferentially treated by prompt endovascular coiling, which is not available in Guadeloupe. Subsequently, patients are transferred to Paris, France mainland, by commercial airplane (6751 km flight) after being managed according to guidelines. This study describes the characteristics, management and outcomes related to these patients.

**Methods:**

Retrospective observational cohort study of 148 patients admitted in intensive care unit for a suspected aSAH and transferred by airplane over a 10-year period (2010–2019).

**Results:**

The median [interquartile range] age was 53 [45–64] years and 61% were female. On admission, Glasgow coma scale was 15 [13–15], World Federation of Neurological Surgeons (WFNS) grading scale was 1 [1–3] and Fisher scale was 4 [2–4]. External ventricular drainage and mechanical ventilation were performed prior to the flight respectively in 42% and 47% of patients. One-year mortality was 16% over the study period. By COX logistic regression analysis, acute hydrocephalus (hazard ratio [HR] 2.34, 95% confidence interval [CI] 0.98–5.58) prior to airplane transfer, WFNS grading scale on admission (HR 1.53, 95% CI 1.16–2.02) and age (OR 1.03, 95% 1.00–1.07) were associated with one-year mortality.

**Conclusion:**

When necessary, transatlantic air transfer of patients with suspected aSAH after management according to local guidelines seems feasible and safe.

**Supplementary Information:**

The online version contains supplementary material available at 10.1186/s12871-024-02532-7.

## Introduction

Worldwide incidence of spontaneous aneurysmal subarachnoid hemorrhage (aSAH) is about 6.1 per 100,000 person-years, depending on the country [1]. Despite advances in the management of this disease [2], early case-fatality remains high, with about 25% of death and a functional dependence in more than one-third of the survivors [3–6]. Aneurysm rebleeding is a life-threatening event that occurs within the first 24 h in 2–13% of patients [7, 8]. Rebleeding is also associated with poor functional recovery in survivors. To reduce the rate of rebleeding, the aneurysm should be secured as early as possible [9]. If possible, coiling should be preferred, and guidelines recommend an early transfer of patients with aSAH to high-volume center (e.g. >35 aSAH case per year) [9].

Guadeloupe is a French West Indies Caribbean island of the Lesser Antilles archipelago. It is one of the 2 French overseas departments and regions with 440,000 inhabitants where neuro endovascular treatment for cerebral aneurysms is not available. It is a fully-fledged French territory therefore compared to other Caribbean destinations, being a French department makes any exchange with France easier from a practical point of view, in terms of currency, laws, travel and accommodation facilities. Furthermore, Caribbean islands do not belong to a common health system. Each island has historic links with European or American mainland, and its own policy of sanitary transfer. To date, in terms of health, there is no agreement between the Caribbean islands; which is why French patients are transferred to France mainland, yet further than the much closer Caribbean islands.

The low incidence of aSAH in the Southern Caribbean population (3.29 per 100,000 person-years) [10], prevent the main hospital of Guadeloupe from meeting the criteria of a high-volume center to appropriately treat aSAH.

To address this issue, for more than 10 years, all patients with aSAH have been transferred as soon as possible to Paris in France mainland, on a commercial flight with specific medical assistance. The transfer is an eight-hour flight and there are two to five commercial flights a day. Similar arrangements have been adopted in Martinique, another French West Indies Island [11]. In addition to international guidelines, to optimize patient’s safety during the flight, our department has developed local guidelines to manage patients prior to the flight.

Here, we report the transatlantic management of aSAH in Guadeloupe over a 10-year period. Our first aim was to describe the characteristics and the management of these patients. In addition, we described the adverse events associated with the transatlantic transfer. Finally, we assessed one-year mortality and identified the related factors.

## Patients and methods

### Setting and study desigsn

This retrospective observational single-center study of patients with SAH by ruptured intracranial aneurysm was carried out at the University Hospital of Guadeloupe, the main and major hospital of the area, with 440 beds. As the most advanced center for the entire Guadeloupe archipelago, all patients with aSAH are managed there. Since 2010, all patients with suspected aSAH are transferred by airplane to one unique high-volume center in Paris, the Rothschild Foundation Hospital where digital subtractive angiography and subsequent coiling is performed. Although neurosurgical clipping is possible at the University Hospital of Guadeloupe, coiling is preferred [2, 11, 12]. Clipping in Guadeloupe is only performed in patients who are not transferred for coiling (e.g. lack of health insurance).

**Fig. 1:**
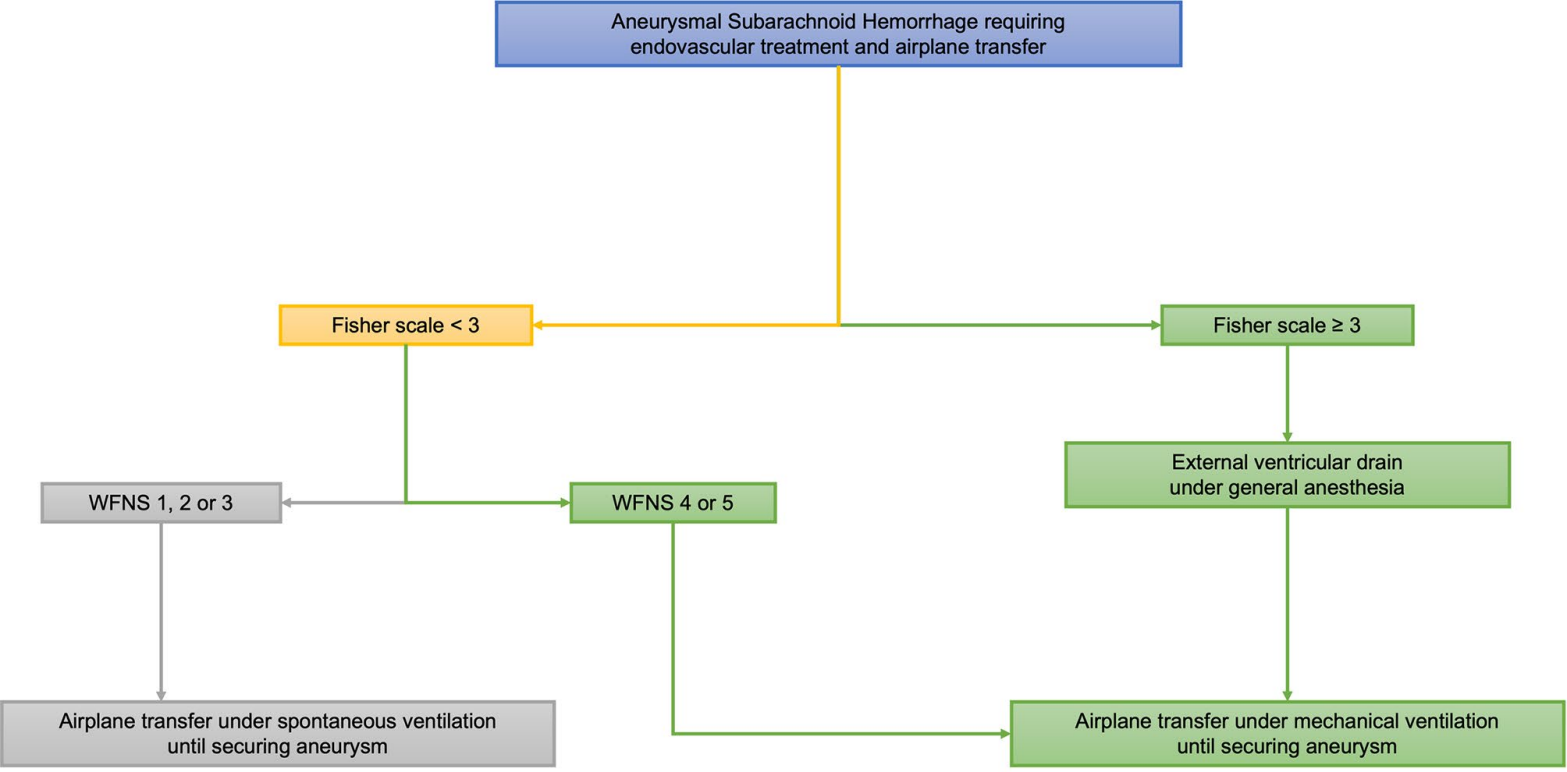
Management of patients with aneurysmal subarachnoid hemorrhage prior to airplane transfer on a commercial flight according to our local guidelines. WFNS, World Federation of Neurological Surgeons

Airplane transfer includes hospital to airport transfer, installation of the patient in the airplane, flight time, exit from the airplane and transfer from airport to the reference center. Once aSAH has been diagnosed and airplane transfer to the reference center in Paris decided, patients are managed until the transfer based on transcranial doppler, clinical assessment and computed tomography, according to guidelines [13]. In addition, to secure and try to prevent complications during the 15-hour long transfer, including an 8-hour flight, additional specific guidelines for airplane transfer of aSAH patients were developed by both teams (University Hospital of Guadeloupe and Rothschild Foundation Hospital), based on our common experience, including a systematic control CT scan performed before flight. Figure 1 summarizes these guidelines (complete guidelines of the University Hospital of Guadeloupe are available in the Supplementary Material). University Hospital of Guadeloupe Guidelines for the management of aneurysmal subarachnoid hemorrhage).

First, because external ventricular drainage (EVD) placement is impossible during the transfer and especially in-flight, EVD was performed under general anesthesia before the flight in patients with a Fisher scale [14] ≥ 3, since these patients are at high risk of developing hydrocephalus, defined by abnormal symptomatic accumulation of cerebrospinal fluid inside the cerebral ventricles and subsequent intracranial hypertension [15, 16]. Of notice, after EVD was performed, these patients remained intubated and mechanically ventilated for the whole transfer. Second, patients with a Fisher scale < 3 but a World Federation of Neurological Surgeons (WFNS) [17] grading scale ≥ 4 were preemptively intubated before the flight because these patients are at high risk of worsening level of consciousness and require endotracheal intubation, which can be difficult due to limited space on a commercial airplane. Our guidelines did not recommend the use of tranexamic acid [18]. Even if interventions before transatlantic transfers (e.g. EVD or intubation) were recommended by our guidelines, the final decision was under the supervision of the intensivist in charge of the patient. The entire transfer was managed by a team consisting of a physician and a nurse with a high expertise in airplane transfer of aSAH patients. The teams providing the transfer have not only received a theoretical training, but also a practical one. These teams have, in addition, carried out at least 50 transfers of ventilated intubated patients.

Continuous intracranial pressure measurement was performed in the ICU prior to the transfer, but measurement was not available during the transfer (monitors used for airplane transfer were not equipped with this function). Therefore, hourly ventricular drainage (about 10 mL) was performed if EVD was productive. If less than 10 mL was evacuated, the transfer team checked if EVD was permeable. If it was, EVD drainage was performed every couple of hours or hourly volume was minored. All these adjustments were done with close supervision of the patient.

On the ventilator, tidal volume was set to 6 mL/kg with plateau pressure < 30cmH_2_O and a respiratory rate so that end tidal CO_2_ remained between 38 and 42 mmHg. Blood pressure was managed in accordance with the last transcranial doppler realized before leaving the ICU, using either norepinephrine or antihypertensive therapies to fit the objectives. Oral nimodipine (60 mg) was administered each 4 h. Sedations were by either propofol (≈ 3 mg/kg/h) or midazolam (≈ 0.1 mg/kg/h) associated with sufentanil (20–30 µg/h). No tranexamic acid was used.

**Fig. 2:**
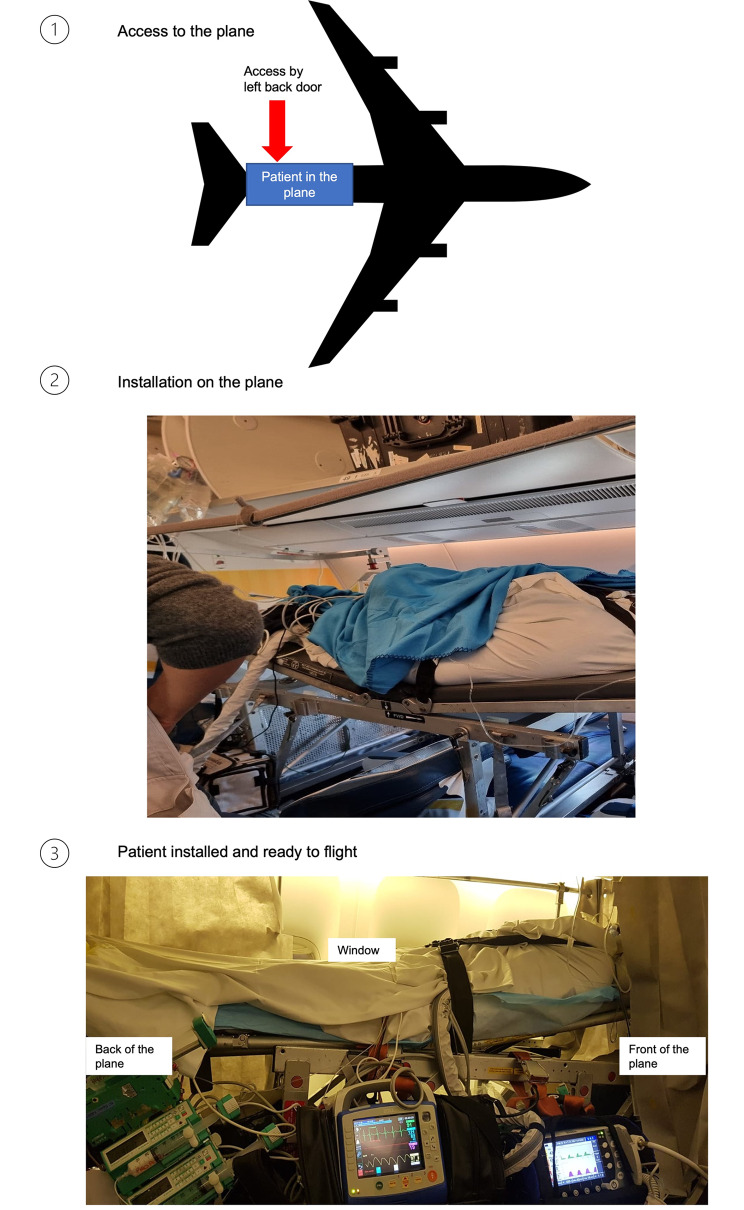
Steps for installation of the patient in the plane. Airplane silhouette is reprinted from [https://upload.wikimedia.org/wikipedia/commons/0/00/Avion_silhouette.svg] under a CC BY license, with permission from [Andreas 06, Public domain, via Wikimedia Commons], original copyright [2006] [19]. Other pictures are author’s personal properties

**Fig. 3:**
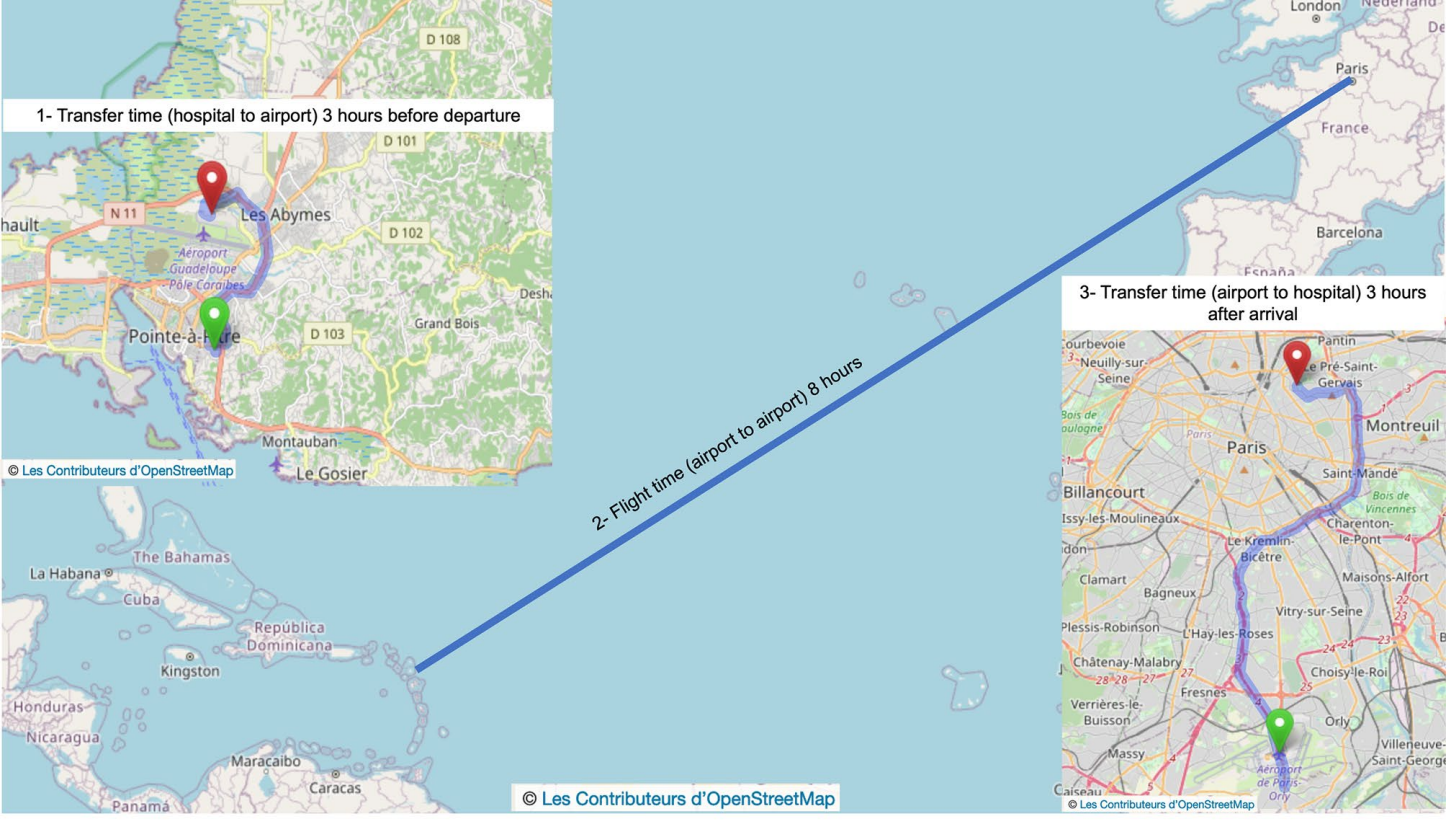
Transfer from University Hospital of Guadeloupe to Rothschild Foundation Hospital (reference center) in Paris. Maps are reprinted from [https://openstreetmap.org] under a CC BY license, with permission from [OpenStreetMapFoundation], original copyright [2018] [20]

University Hospital of Guadeloupe is 8 km distant from the airport. Using a specific elevator positioned on the left back door of the plane, transferred patients are boarded three hours before the scheduled departure hour and other passengers. Six seats are required to install the patient supine on a flat stretcher, with the head at least 30° elevated, in the direction of the cockpit. Two medical oxygen kits (1,27 × 10^7^ Pascals, 3250 L each) have previously been installed. All devices are then fixed for the flight (monitor, respirator, electric syringe pumps) (Fig. 2). Flight time is about 8 h (6751 km, Fig. 3). On arrival, patients are disembarked after passengers and transferred to the reference center (22 km from the airport). Total transfer time door to door from the University Hospital of Guadeloupe to the reference center is 14 to 16 h.

Despite the fact the environment is less adapted to care, we opted for airplane transfers on commercial flights rather than ambulance flights because of the high number of daily commercial flights (three to five) and because ambulance flights should first come from mainland France, which would delay the transfer by at least 12 h. Of notice the cost of an airplane transfer in a commercial flight is about 14.000 euros, which is much lower than the cost of a medevac on an ambulance flight that is approximately 140.000 euros Two scheduled airlines operate sanitary airplane transfers. Minimal timing to organize an airplane transfer is 6 h (management of the patient, medical team for transport, availability on the flight or requisition by disembarking passengers). Therefore, if subarachnoid hemorrhage is diagnosed less than 6 h before the last flight, transfer is postponed for a day and so management in the reference center is also delayed, which increases the risk of complications (rebleeding, vasospasm, seizure…etc).

### Patient selection

Consecutive patients admitted between January 1st 2010 and December 31st 2019 with non-traumatic subarachnoid hemorrhage confirmed by computed tomography or magnetic resonance imaging, were included. Patients with arteriovenous malformation, age < 18 years old and those who were not transferred to mainland France (no health insurance or treatment withholding) were excluded from the study. For patients with several admissions for non-traumatic subarachnoid hemorrhage, only the first stay was included in the analysis. Of note, since cerebral angiography was not available at the University Hospital of Guadeloupe, patients with non-traumatic subarachnoid hemorrhage in whom no arteriovenous malformation or aneurysm was seen on brain angio-computed tomography were assumed to be caused by an aneurysm and were subsequently airplane transferred for digital subtraction angiography and possible coiling.

### Data collection

We collected the following data: age, gender, previous arterial hypertension, day of bleeding, clinical characteristics at hospital admission (Glasgow coma scale, neurological deficit, WFNS grading scale and Fisher scale). We also collected the characteristics of the aneurysm (size, number, and location), the complications before securing (acute hydrocephalus based on computed tomography, intracranial hypertension based either on abnormal transcranial doppler defined by decrease of end-diastolic velocity < 20 cm/s and /or increase of pulsatility index > 1.20 or abnormal computed tomography suspected on disappearance of the cortical furrows or filling of the mesencephalic cisterns or disappearance of the ventricles, rebleeding) and the management before transatlantic airplane transfer (EVD, tracheal intubation and subsequent mechanical ventilation). We finally collected complications that occurred during the transatlantic airplane transfer and during the first day at the reference center in France mainland, 30-day and one-year mortality, modified Rankin Scale score [21] and Glasgow outcome scale [22] at discharge from the hospital.

The modified Rankin Scale is a tool used to classify the degree of handicap in stroke [21]. The scale is organized in six levels, with one more for patients who died: 0 is no symptoms; 1 is no significant disability, able to carry out all usual activities, despite some symptoms; 2 is slight disability, able to look after one’s own affairs without assistance, but unable to carry out all previous activities; 3 is moderate disability, requires some help, but able to walk unassisted; 4 is moderately severe disability, unable to attend to own bodily needs without assistance, and unable to walk unassisted; 5 is severe disability, requires constant nursing care and attention, bedridden, incontinent; 6 is when the patient has died.

The Glasgow outcome scale is used after brain damage to assess persisting disability [22]. The scale is organized in five levels.1 is when the patient has died. 2 is Persistent vegetative state: severe damage with prolonged state of unresponsiveness and a lack of higher mental functions. 3 is severe disability: severe injury with permanent need for help with daily living. 4 is moderate disability: no need for assistance in everyday life, employment is possible but may require special equipment. 5 is low disability: light damage with minor neurological and psychological deficits. According to previous publications [23] we considered a good neurological outcome at hospital discharge as values of modified Rankin Scale from 0 to 3 and as values of Glasgow Outcome Scale from 4 to 5.

Data collected were those of patients admitted between January 2010 and December 2019. Authors never had access to information that could identify individual participants during or after data collection.

### Statistical analysis

Analyses were performed using Prism V9.4.1 (GraphPad software, Boston, MA) and R statistical software, version 4.0.4 (available online at http://www.r-project.org/). Quantitative variables were described as median [interquartile range] and were compared between groups using the non-parametric Wilcoxon rank-sum test. Qualitative variables were described as n (%) and were compared between groups using Fisher’s exact test. To evaluate a potential change in patient’s mortality and severity across the 10-year study period, we arbitrarily divided it into two 5-year study periods, 2010–2014 and 2015–2019. Potential changes in mortality rates over the study period were analyzed using a Fisher’s exact test. Potential changes of severity over time were analyzed using a Wilcoxon rank-sum test. Cox proportional-hazard regression analysis was used to identify the variables significantly associated with one-year mortality. Variables yielding *p*-values < 0.20 in the univariate analyses were considered. Variables used to calculate scores (e.g. WFNS) were not entered in the model. In addition, we did not enter variables related to interventions before airplane transfer or variables determined by the airplane transfer (e.g. intubation and external ventricular drain). Eventually, five variables were entered in the multivariate analysis. We performed backward selection on the model, stopping when the Akaike information criterion reached its minimum. Results were reported as Hazard ratios (HR), with their 95% confidence interval (95% CI).

Kaplan-Meier overall survival curves up to one year were separately computed for relevant variables and were compared using log-rank tests. No imputation was performed for missing values. A *p*-value less than 0.05 was considered significant.

## Results

**Fig. 4:**
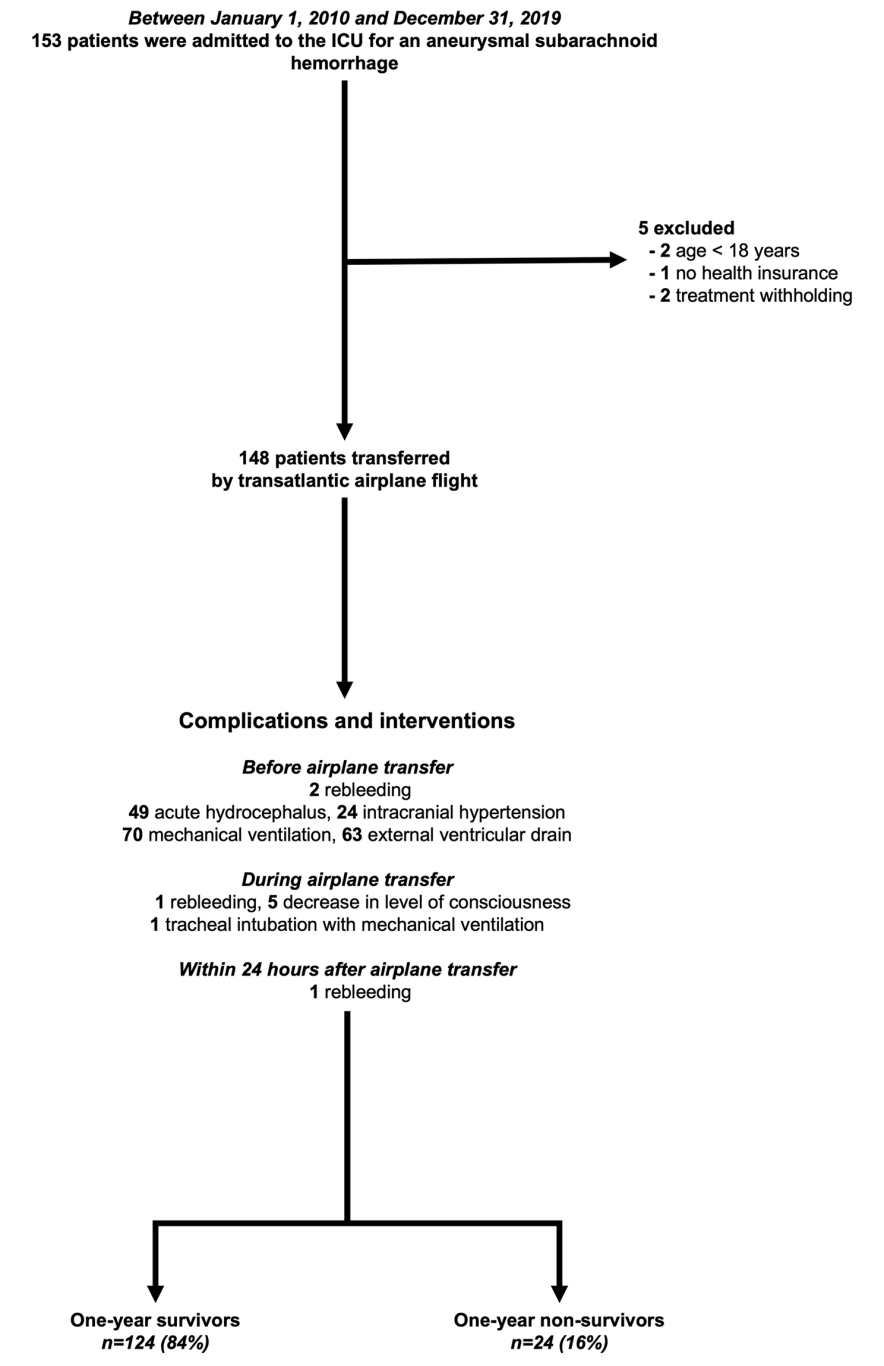
Study flow chart and complications during airplane transfer

**Fig. 5:**
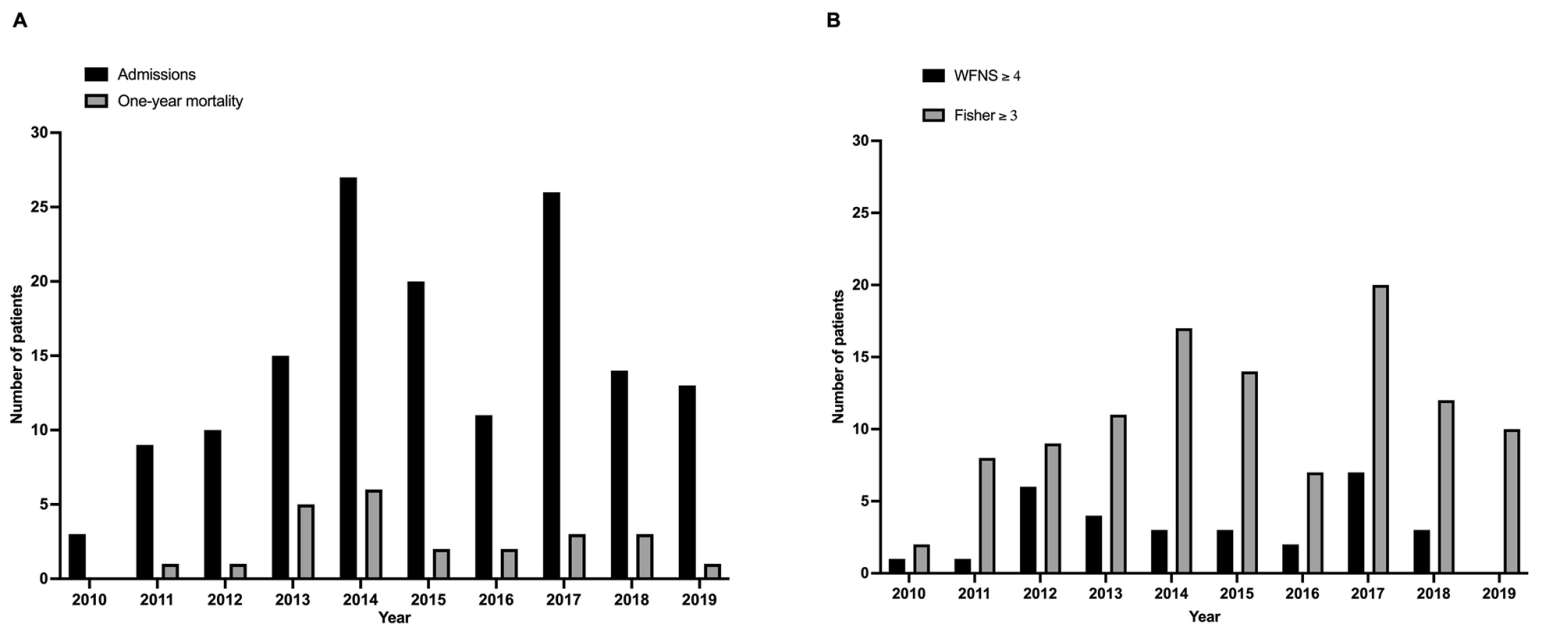
Overview of mortality and severity over the study period. (**A**) Annual number of admission and deaths over the study period. (**B**) Number of patients with a World Federation of Neurological Surgeons grading scale ≥ 4 and a Fisher scale ≥ 3 over the study period

Figure 4 shows the study flow chart. Over the study period, 153 patients were admitted for a suspected aSAH with clinical symptoms of hemorrhage. Among them, two patients were < 18 years old and three patients were not transferred to Paris; two patients because treatment withholding was decided due to a poor prognosis and one because he did not have health insurance. For the last patient, neurosurgical clipping was performed. Eventually, 148 patients were enrolled in the analysis. Figure 5 is an overview of mortality and severity of the disease along the 10 years study period.

### Patient characteristics

Table 1 shows the characteristics of the study population. The median age was 53 [45–63] years, 90 patients (61%) were female and 69 (47%) patients had arterial hypertension. On admission, Glasgow coma scale was 15 [13–15], WFNS grading scale was 1 [1–3] and Fisher scale was 4 [2–4]. Between 2010 and 2014 and 2015–2019, the proportion of patients with a WFNS grading scale ≥ 4 and a Fisher scale ≥ 3 did not differ (*p* = 0.952 and *p* = 0.421, respectively) (Fig. 5B). After digital subtraction angiography, 16 patients did not have aneurysm and were classified *sine materiae*. In the remaining 132 patients who had a proven aSAH, most of the aneurysms were located on the carotid system (87%, *n* = 115/132), with 41% (*n* = 61/132) on the anterior communicating artery. Fifteen patients (10%) had more than one aneurysm.

**Table 1:** Characteristics, management and outcome of the patients

	*n* = 148
Age, *years*	53 [45–64]
Gender, Woman, *n (%)*	90 (61)
Hypertension, *n (%)*	69 (47)
**Characteristics on admission**	
Glasgow Coma Scale	15 [13–15]
Glasgow Coma Scale 15, *n (%)*	82 (55)
Glasgow Coma Scale 13–14, *n (%)*	32 (22)
Glasgow Coma Scale 7–12, *n (%)*	22 (15)
Glasgow Coma Scale 3–6, *n (%)*	12 (8)
Neurological deficit, *n (%)*	40 (27)
WFNS grading scale^a^	1 [1–3]
WFNS 1, *n (%)*	82 (55)
WFNS 2, *n (%)*	23 (16)
WFNS 3, *n (%)*	9 (6)
WFNS 4, *n (%)*	22 (15)
WFNS 5, *n (%)*	12 (8)
Fisher scale	4 [2–4]
Fisher 1, *n (%)*	17 (11)
Fisher 2, *n (%)*	21 (14)
Fisher 3, *n (%)*	29 (20)
Fisher 4, *n (%)*	81 (55)
**Characteristics of the aneurysm**	
Number of aneurysms > 1, *n (%)*	15 (10)
Aneurysm size, *mm*^b^	5.4 [4.1–8.0]
Location of aneurysm	
Anterior artery (cerebral and communicating), *n (%)*	61 (41)
Internal carotid artery, *n (%)*	29 (20)
Middle cerebral artery, *n (%)*	19 (13)
Basilar artery, *n (%)*	9 (6)
Posterior inferior cerebellar artery, *n (%)*	8 (5)
Pericallosal artery, *n (%)*	6 (4)
*Sine materiae* subarachnoid hemorrhage, *n (%)*	16 (11)
**Complications before securing**	
Acute hydrocephalus, *n (%)*	49 (33)
Intracranial hypertension, *n (%)*	24 (16)
Rebleeding, *n (%)*	4 (3)
**Interventions before transatlantic transfer**	
Tracheal intubation with mechanical ventilation, *n (%)*	70 (47)
External ventricular drain, *n (%)*	63 (42)
**Time from bleeding to coiling**, ***days***^c^	2 [2–3]
**Main outcomes**	
30-day mortality, *n (%)*	13 (9)
One-year mortality, *n (%)*	24 (16)
Glasgow outcome scale at hospital discharge^d^	5 [3–5]
Glasgow outcome scale 1–3, *n (%)*	34 (34)
Glasgow outcome scale 4–5, *n (%)*	65 (66)
Modified Rankin scale at hospital discharge^e^	2 [1–5]
Modified Rankin scale 0–3, *n (%)*	69 (69)
Modified Rankin scale 4–6, *n (%)*	31 (31)

Quantitative variables are described as median [interquartile range] and qualitative variables are described as frequency (percentages)

^a^WFNS, World Federation of Neurological Surgeons

^b^*n* = 98

^c^*n* = 133

^d^*n* = 99

^e^*n* = 100

### Transfer from Guadeloupe to Paris

Prior to airplane transfer, intubation and subsequent mechanical ventilation was instituted in 47% (*n* = 70) of patients. Among them, mechanical ventilation was instituted to perform EVD placement in 41% of patients (*n* = 61), who remained intubated during the whole transfer; because of a WFNS grading scale of ≥ 4 in 2% of patients (*n* = 3) and based on the physician’s decision in the remaining 4% (*n* = 6). According to our airplane transfer guideline (Fig. 1), EVD was performed because of acute hydrocephalus in 27% (*n* = 40) and because of a Fisher scale ≥3 without acute hydrocephalus in the remaining 15% (*n* = 22). One patient did not fit these criteria and still had an EVD. Among patients suffering from intracranial hypertension, 8 (5%) did not have acute hydrocephalus. Of notice, rebleeding was observed in two patients before the flight (systematic control CT scan performed before flight).

During airplane transfer, six non-intubated patients worsened their level of consciousness, which occurred mostly during take-off. In five patients, the Glasgow coma scale remained > 8, these patients did not require tracheal intubation. However, one patient rebled (confirmed by regular CT scan performed after arrival) and required endotracheal intubation during the flight.

Upon arrival at the Rothschild Foundation Hospital in Paris, rebleeding occurred in one patient within the first 24 h. Digital subtraction angiography was performed in all patients. One or more aneurysms were found in 132 of the 148 patients, all 132 patients underwent endovascular coiling. The time from bleeding to securing the aneurysm was 2 [2–3] days. Digital subtraction angiography did not show an aneurysm or other source of subarachnoid hemorrhage in 16 patients (11%), who were classified as *sine materiae* subarachnoid hemorrhage, after a new digital subtraction angiography check within 10 days (none of these cases showed an aneurysm on the second angiography).

### Mortality and functional outcome

Thirty-day mortality was 9% (*n* = 13) and one-year mortality was 16% (*n* = 24). One-year mortality was not different between the 2010–2014 period (20%, *n* = 13) and the 2015–2019 period (18%, *n* = 11, *p* > 0.999) (Fig. 5A). Table 2 shows factors associated with one-year mortality. By COX logistic regression, three factors were independently associated with one-year mortality: acute hydrocephalus (HR 2.34, 95% CI 0.98–5.58) prior to airplane transfer, WFNS grading scale on admission (HR 1.53, 95% CI 1.16–2.02) and age (OR 1.03, 95% 1.00–1.07).

**Table 2:** Factors associated with one-year mortality: univariate and multivariate analysis

	Univariate analysis *(n = 148)*			*Multivariate analysis (n = 148)*	
	One-year survivors *n* = 124	One-year non-survivors *n* = 24	*P*-value^a^	Hazard ratio (95% confidence interval)	*P*-value^b^
Age, *years*	53 (45–60)	67 (51–71)	0.006	1.03 (1.00–1.07)	0.045
Gender, Woman, *n (%)*	76 (61)	14 (58)	0.822		
Hypertension, *n (%)*	57 (46)	12 (50)	0.824		
Characteristics on admission					
Glasgow Coma Scale	15 (13–15)	12 (8–14)	< 0.001		
Neurological deficit, *n (%)*	30 (24)	10 (42)	0.085		
WFNS grading scale	1 (1–2)	4 (2–4)	< 0.001	1.53 (1.16–2.02)	0.003
Fisher scale	4 (2–4)	4 (3–4)	0.030		
Complications before securing					
Acute hydrocephalus, *n (%)*	34 (27)	15 (63)	0.002	2.34 (0.98–5.58)	0.054
Intracranial hypertension, *n (%)*	16 (11)	8 (33)	0.029		
Rebleeding, *n (%)*	3 (2)	1 (4)	0.511		
Interventions before transatlantic transfer					
Mechanical ventilation, *n (%)*	52 (42)	18 (75)	0.004		
External ventricular drain, *n (%)*	47 (38)	16 (67)	0.013		
Time from bleeding to coiling, *days*	2 (2–3)	2 (2–3)	0.385		
Complications during the flight, n (%)	6 (5)	1 (4)	> 0.999		

Quantitative variables are described as median [interquartile range] and qualitative variables are described as frequency (percentages)

WFNS, World Federation of Neurological Surgeons

^a^*p* values were calculated using Wilcoxon rank-sum test and Fisher’s exact test

^b^*p* values were calculated using Cox proportional-hazard model

**Fig. 6:**
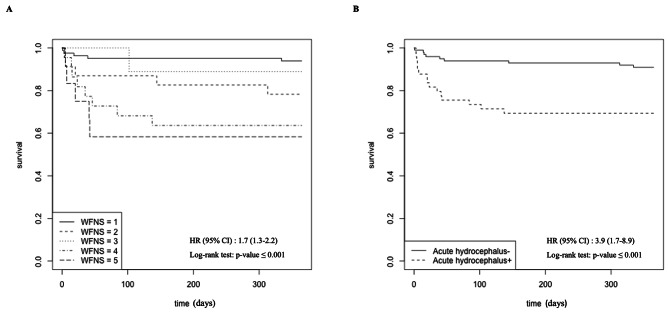
Kaplan–Meier survival estimates during the one year following bleeding. (**A**) According to the World Federation of Neurological Surgeons grading scale. (**B**) According to the presence or absence of acute hydrocephalus before transfer

Figure 6 shows Kaplan–Meier survival estimates according to the World Federation of Neurological Surgeons grading scale and the presence or absence of acute hydrocephalus before transfer. By 30-day, all patients were not returned to Guadeloupe. Among deceased patients, 58% (*n* = 14) died in Paris and 42% (*n* = 10) died in Guadeloupe. No patients experienced complications or died during the return airplane journey from Paris to Guadeloupe.

The proportion of patients with good neurological outcomes at hospital discharge defined by modified Rankin Scale 0 to 3 and Glasgow Outcome Scale 4 to 5 was respectively 69% (*n* = 69) and 66% (*n* = 65).

## Discussion

The main results of our study are that transatlantic airplane transfer on commercial flight of patients with suspected aSAH with prior management according to local guidelines (1) is feasible without excessive delay, (2) is safe, as suggests the small number of complications that occurred during the transfer, (3) is associated with a 16% one-year mortality, which is comparable to what large cohorts of non-airplane transferred aSAH patients reported [2, 24].

Aneurysmal subarachnoid hemorrhage is a life-threatening disease with a high mortality and morbidity rate [24, 25]. Guidelines recommend that “low-volume hospitals (e.g. <10 aSAH cases per year) should consider early transfer of patients with aSAH to high-volume centers (e.g. >35 aSAH cases per year) with experienced cerebrovascular surgeons, endovascular specialists and multidisciplinary neuro-intensive care services” [9]. Given the low incidence of aSAH in Guadeloupe, patients should be transferred to a high-volume center. Mejdoubi & al. report patients transferred for coiling to a center in France mainland had a lower mortality rate and a better functional outcome than patients who underwent neurosurgical treatment in Martinique island [11]. Of notice, in this series of airplane transferred patients, rebleeding was low, as it was in our cohort.

Due to the lack of treaties about common health system between Caribbean islands and.

related to strong historic links between Guadeloupe and mainland France, most families in Guadeloupe have at least one relative living in mainland France. This is inasmuch the case as Guadeloupe is a French speaking territory, living and communicating could be more difficult for relatives who want to stay close to the patient in an English or Spanish-speaking Caribbean island than in France mainland. Obviously, this strategy has a high cost. The ultimate goal remains a high-volume aSAH center in the Lesser Antilles area.

In our study, we report few complications during the flight. Because patients are supine on a flat stretcher in the airplane, with the head at least 30° elevated, in the direction of the cockpit (Fig. 2), the decrease in the level of consciousness occurred mostly during take-off, which, combined with acceleration, may worsen cerebral perfusion [26]. Thus, during transfer, continuous monitoring of intracranial pressure should be performed for all patients with EVD, and particularly in those intubated. In addition, autonomic cardiovascular control is disturbed during acute exposure to an air pressure similar to the air pressure encountered inside pressurized commercial airplanes, about 750 hPa [27]. The low incidence of complications we reported during the flight might be explained by the expertise of the medical team that supervised the transfer. It may also be explained by the patient management upstream of the flight (i.e. prophylactic EVD and endotracheal intubation). These local guidelines were actually the fruit of the experience gained by the University Hospital of Guadeloupe and the Rothschild Foundation Hospital in their collaboration.

No patient was not transferred because of lack of availability on the plane and disembarking passengers is sometimes necessary in order to prioritize the sanitary transfers. Transfer can be delayed to the next day (time too short or no more plane on departure), then, patients are managed and closely monitored in ICU to prevent complications (EVD placement, transcranial doppler, CT scan, prevention of acute hydrocephalus and intracranial hypertension, early administration of Nimodipine.)

With a 16% one-year mortality rate, our strategy was associated with a similar outcome to large cohorts of aSAH patients (22 to 23% one-year mortality) [2, 24]. Overall, the prognosis of patients at discharge of hospital (Glasgow outcome scale and modified Rankin scale) appears to be similar in the range of the health-related quality of life that is reported in patients who were not air transferred [2, 5, 24, 28]. Identification of factors associated with a poor outcome may help improve the management and possibly the outcome. Unfortunately, among the three factors identified, two are not amenable (WFNS grading scale and age at the time of diagnosis). Fortunately, the third one, acute hydrocephalus, can be amended by early EVD which may help reduce intracranial pressure.

A major strength of our study is that it is the largest cohort of aSAH transferred by airplane on long haul flights. To date, few studies have reported long duration airplane transfers [29], and particularly in aSAH [11]. None of them has reported one-year mortality. Our study has limitations. First, because of the retrospective and monocentric design of the study, these results should be interpreted with caution. Our findings need to be confirmed by future prospective controlled studies. Second, the retrospective design prevents us from assessing the long-term dependency and quality of life among survivors. Third, a precise description of physiologic parameters during airplane transfer is lacking. Fourth, with the endpoint of mortality, vasospasm or symptomatic vasospasm, delayed ischemic deficits and other complications associated with transport while in Paris could not be accounted for. Fifth, although this is the largest study on the topic, our sample size remains limited. Finally, a control group of patients with similar characteristics who were not airplane transferred is also missing. However, such group is not available since all our patients with aSAH were air transferred.

## Conclusion

In the event of geographical obstacles and constraints, such as insularity, subarachnoid hemorrhage management, involving airplane transfer on long haul commercial flight appears feasible and safe. These results should be interpreted with caution considering the retrospective and monocentric nature of the study. The low level of per flight complications observed may be explained by our local guidelines to secure as much as possible the airplane transfers ahead of the flight. This strategy was associated with a 16% one-year mortality, which is comparable to what is reported in aSAH patients managed without air transfer. Nevertheless, these findings need to be consolidated by prospective and controlled studies.

### Electronic supplementary material

Below is the link to the electronic supplementary material.

Supplementary Material 1

Supplementary Material 2

## Data Availability

The datasets used and/or analyzed during the current study are available from the corresponding author on reasonable request.

## Abbreviations

aSAH: aneurysmal subarachnoid hemorrhage
EVD: external ventricular drainage
HR: hazard ratios
ICU: intensive care unit
WFNS: World Federation of Neurological Surgeons
CI: confidence interval

## References

[1] Etminan N, Chang HS, Hackenberg K, de Rooij NK, Vergouwen MDI, Rinkel GJE (2019). Worldwide Incidence of Aneurysmal Subarachnoid Hemorrhage according to Region, Time Period, blood pressure, and Smoking Prevalence in the Population: a systematic review and Meta-analysis. JAMA Neurol.

[2] Molyneux AJ, Kerr RS, Yu LM, Clarke M, Sneade M, Yarnold JA (2005). International subarachnoid aneurysm trial (ISAT) of neurosurgical clipping versus endovascular coiling in 2143 patients with ruptured intracranial aneurysms: a randomised comparison of effects on survival, dependency, seizures, rebleeding, subgroups, and aneurysm occlusion. Lancet.

[3] Claassen J, Park S (2022). Spontaneous subarachnoid haemorrhage. Lancet.

[4] Goldberg J, Schoeni D, Mordasini P, Z’Graggen W, Gralla J, Raabe A (2018). Survival and Outcome after Poor-Grade Aneurysmal Subarachnoid Hemorrhage in Elderly patients. Stroke.

[5] Hua X, Gray A, Wolstenholme J, Clarke P, Molyneux AJ, Kerr RSC (2021). Survival, dependency, and Health-related quality of life in patients with ruptured intracranial aneurysm: 10-Year follow-up of the United Kingdom Cohort of the International Subarachnoid Aneurysm Trial. Neurosurgery.

[6] Ironside N, Buell TJ, Chen CJ, Kumar JS, Paisan GM, Sokolowski JD (2019). High-Grade Aneurysmal Subarachnoid Hemorrhage: predictors of functional outcome. World Neurosurg.

[7] Naidech AM, Janjua N, Kreiter KT, Ostapkovich ND, Fitzsimmons BF, Parra A (2005). Predictors and impact of aneurysm rebleeding after subarachnoid hemorrhage. Arch Neurol.

[8] Ohkuma H, Tsurutani H, Suzuki S (2001). Incidence and significance of early aneurysmal rebleeding before neurosurgical or neurological management. Stroke.

[9] Connolly ES, Rabinstein AA, Carhuapoma JR, Derdeyn CP, Dion J, Higashida RT (2012). Guidelines for the management of aneurysmal subarachnoid hemorrhage: a guideline for healthcare professionals from the American Heart Association/american Stroke Association. Stroke.

[10] Schertz M, Mehdaoui H, Hamlat A, Piotin M, Banydeen R, Mejdoubi M (2016). Incidence and mortality of spontaneous subarachnoid hemorrhage in Martinique. PLoS ONE.

[11] Mejdoubi M, Schertz M, Zanolla S, Mehdaoui H, Piotin M (2018). Transoceanic Management and Treatment of Aneurysmal Subarachnoid Hemorrhage: a 10-Year experience. Stroke.

[12] Hoh BL, Ko NU, Amin-Hanjani S, Chou S-Y, Cruz-Flores S, Dangayach NS (2023). 2023 Guideline for the management of patients with Aneurysmal Subarachnoid Hemorrhage: a Guideline from the American Heart Association/American Stroke Association. Stroke.

[13] Greenberg SM, Ziai WC, Cordonnier C, Dowlatshahi D, Francis B, Goldstein JN (2022). 2022 Guideline for the management of patients with spontaneous intracerebral hemorrhage: a Guideline from the American Heart Association/American Stroke Association. Stroke.

[14] Fisher CM, Kistler JP, Davis JM (1980). Relation of cerebral vasospasm to subarachnoid hemorrhage visualized by computerized tomographic scanning. Neurosurgery.

[15] Demirgil BT, Tugcu B, Postalci L, Guclu G, Dalgic A, Oral Z (2003). Factors leading to hydrocephalus after aneurysmal subarachnoid hemorrhage. Minim Invasive Neurosurg.

[16] Rekate HL (2009). A contemporary definition and classification of hydrocephalus. Semin Pediatr Neurol.

[17] Report of World Federation of Neurological Surgeons Committee on a Universal Subarachnoid Hemorrhage Grading Scale (1988). J Neurosurg.

[18] Post R, Germans MR, Tjerkstra MA, Vergouwen MDI, Jellema K, Koot RW (2021). Ultra-early tranexamic acid after subarachnoid haemorrhage (ULTRA): a randomised controlled trial. Lancet.

[19] 06 A. Silhouette of an airplane. In: Silhouette A, editorWikimedia Commons. 2006. https://commons.wikimedia.org/wiki/File:Avion_silhouette.svg

[20] openstreetmap.org h. cartographer OpenStreetMap. https://openstreetmap.org: OpenStreetMap; 2018.

[21] van Swieten JC, Koudstaal PJ, Visser MC, Schouten HJ, van Gijn J (1988). Interobserver agreement for the assessment of handicap in stroke patients. Stroke.

[22] Jennett B, Bond M (1975). Assessment of outcome after severe brain damage. Lancet.

[23] Rangaraju S, Haussen D, Nogueira RG, Nahab F, Frankel M (2017). Comparison of 3-Month Stroke disability and quality of Life across Modified Rankin Scale categories. Interv Neurol.

[24] Schatlo B, Fung C, Stienen MN, Fathi AR, Fandino J, Smoll NR (2021). Incidence and outcome of Aneurysmal Subarachnoid Hemorrhage: the Swiss study on Subarachnoid Hemorrhage (Swiss SOS). Stroke.

[25] van Gijn J, Kerr RS, Rinkel GJ (2007). Subarachnoid haemorrhage. Lancet.

[26] Ercan E (2021). Effects of aerospace environments on the cardiovascular system. Anatol J Cardiol.

[27] Sevre K, Bendz B, Rostrup M (2002). Reduced baroreceptor reflex sensitivity and increased blood pressure variability at 2400 m simulated cabin altitude. Aviat Space Environ Med.

[28] van Donkelaar CE, Bakker NA, Birks J, Veeger N, Metzemaekers JDM, Molyneux AJ (2019). Prediction of Outcome after Aneurysmal Subarachnoid Hemorrhage. Stroke.

[29] Lebreton G, Sanchez B, Hennequin JL, Resiere D, Hommel D, Leonard C (2012). The French airbridge for circulatory support in the Carribean. Interact Cardiovasc Thorac Surg.

