# Supplementary material for: One-year survival of aneurysmal subarachnoid hemorrhage after airplane transatlantic transfer – a monocenter retrospective study
Source: BMC Anesthesiol. 2024 Apr 12;24:140. doi: 10.1186/s12871-024-02532-7 (PMC11010355; doi:10.1186/s12871-024-02532-7)
Supplement: Supplementary file 2 — Supplementary Material 2 [file 12871_2024_2532_MOESM2_ESM.docx]

| UNIVERSITY HOSPITAL OF GUADELOUPE | |
| --- | --- |
| **GUIDELINES FOR THE MANAGEMENT OF ANEURYSMAL SUBARACHNOID HEMORRHAGES** | Ref. Internal: **--- /---/0000/--/2021**  Ref. HAS  **Data sheet**  Transmitter:  Page: 1/15 |

# Purpose and scope

This protocol concerns the management of patients suffering from subarachnoid hemorrhages due to aneurysmal rupture hospitalized in the Intensive Care Unit of the University Hospital of Guadeloupe.

# Responsibilities

This protocol applies under the responsibility of the Head of the Critical Care Division, the Heads of Department and the Senior Doctors practicing within the establishment.

# Definition and abbreviations

TCD: Transcranial Doppler

EVD: External Ventricular Drainage

SAH: Subarachnoid Hemorrhage

MAP : Mean Arterial Pressure

ICH: Intracranial Hypertension

CT: Computed-Tomography

WFNS: World Federation of Neurological Surgeons

# Objectives

Establish a reference document for the management of patients suffering from subarachnoid hemorrhages due to aneurysmal rupture hospitalized in the Intensive Care Unit of the University Hospital of Guadeloupe.

| - Project 🞎 Controlled | | |
| --- | --- | --- |
| Editors: | Verifier: | Approver: DQCV |
| Name: Dr F. ARDISSON, Dr F. MARTINO,  A. SUREL  Date: 10/01/2021 Visa: | Name: Date: Visa: | Name: Date: Visa: |
| Centre Hospitalier Universitaire Pointe à Pitre / Abymes Route de Chauvel 97159 Pointe à Pitre Cedex | | |

# Description and execution of the process

- 1. **Introduction**

Stroke is the third leading cause of death in France after cancer and cardiovascular disease (1,2).

There are 15% of hemorrhagic strokes, including subarachnoid hemorrhage (SAH) (1-7% of strokes) (3,4).

SAHs are mostly secondary to aneurysmal rupture. Rapid etiological diagnosis is very important because it conditions the therapeutic management of these cerebral hemorrhages.

Indeed, SAH of aneurysmal origin requires early interventional neuroradiological management in a reference center.

The occurrence of early complications that increase morbidity and mortality must lead to the most appropriate management as soon as possible (5-8).

- 1. **Directions**

Subarachnoid hemorrhages due to aneurysmal rupture

- 1. **Relevant personnel**

This protocol applies to doctors, interns, nurses working in the Intensive Care Unit of the University Hospital of Guadeloupe.

- 1. **Subarachnoid hemorrhages due to aneurysmal rupture**

## Definition

Subarachnoid hemorrhages (SAH) are the eruption of blood into the subarachnoid space (9).

They represent a diagnostic and therapeutic emergency.

Mortality remains high and estimated at between 20 and 45% in Europe in the first hours of onset of SAH (10,11).

Several risk factors have been identified as being significantly associated with the occurrence of meningeal hemorrhage: (5, 12,13)

- Female sex
- Tobacco
- high blood pressure
- Alcohol consumption > 150 g/week

## Clinical

The main suggestive sign is an unusual headache with a sudden onset. The intensity of this headache combined with its persistence over time contribute to this clinical suspicion (5, 10, 12, 13).

Other clinical manifestations may be present: coma, convulsions, vomiting, nausea, paralysis of the oculomotor nerves, etc. etc. (5, 10, 12,13).

Meningeal syndrome is not constant, and its onset is often delayed compared to headache (5, 10,13).

An international clinical rating scale for SAH has been established by the World Federation of Neurological Surgeons (14):

| Rank | Score de Glasgow | Motor deficit |
| --- | --- | --- |
| I | 15 | Absent |
| II | 13–14 | Absent |
| III | 13–14 | Present |
| IV | 7–12 | Present or absent |
| V | 3–6 | Present or absent |

## Etiologies

About 85% of subarachnoid hemorrhages are secondary to a ruptured brain aneurysm. Of the remaining 15%, 10% are idiopathic and 5% are related to different causes such as arterial dissections, arteriovenous malformations or fistulas, and pituitary apoplexy (9).

Most intracranial aneurysms are found on the anterior part of the Willis polygon. In descending order of location, we find (15,16):

- the anterior communicating artery (30%),
- the posterior communicating artery (25%),
- middle cerebral artery (20%)
- internal carotid termination (7.5%),
- cerebral pericallosal artery (4.5%),
- the termination of the basal trunk (7 to 10%),
- posterior inferior cerebellar artery (3-4%),


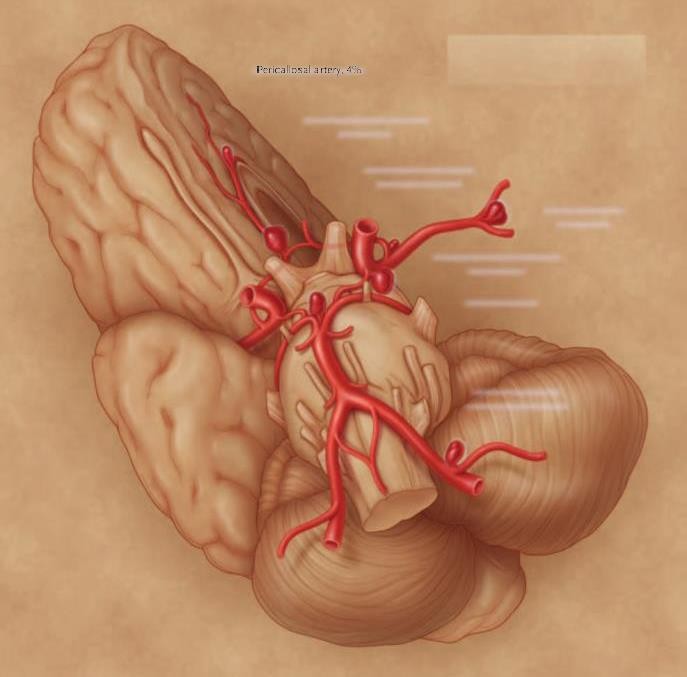

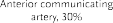

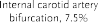

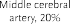

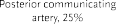

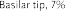

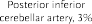

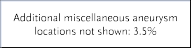


## Positive diagnosis

Clinical suspicion of SAH should require an urgent **non-injected CT scan of the brain** (5,12).

Spontaneous hyperdensity visible in the grooves is a sign of the diagnosis (12):


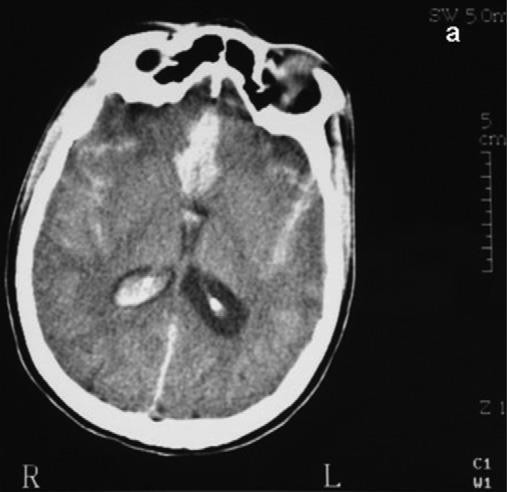


Fisher Subarachnoid Hemorrhage 4

In addition to confirming the diagnosis and allowing an initial assessment of complications, this CT scan will make it possible to classify the meningeal hemorrhage according to the Fisher (5, 12,17) and modified Fisher (26) classifications:

| Fisher's Grade | Scannographic aspect |
| --- | --- |
| 1 | Absence of blood |
| 2 | Deposits less than 1 mm thick |
| 3 | Deposits more than 1 mm thick |
| 4 | Parenchymal hematoma or ventricular hemorrhage |

Fisher CM, Kistler JP, Davis JM. Relation of cerebral vasospasm to subarachnoid hemorrhage visualized by computerized tomographic scanning. Neurosurgery 1980; 6(1):1-9


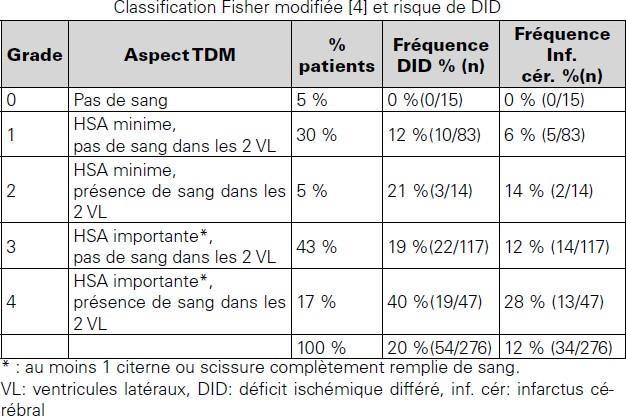


Fauvage, Bertrand, et al. "MANAGEMENT OF SUBARACHNOID HEMORRHAGE (HSA) BY ANEURYSM RUPTURE."

Cerebral **CT angiography** is used as a first-line procedure to establish the etiological diagnosis (5).

Because of the seriousness of some forms of SAH, MRI is not the standard of care, unlike CT scans.

Lumbar puncture is performed only if there is a discrepancy between the clinic and the imaging. The presence of a xanthochromic supernatant after centrifugation indicates the diagnosis (12).

## Main complications

- - - 1. **Rebleeding**

Rebleeding is among the most serious and feared complications. It is linked to significant morbidity and mortality in the range of 20 to 70% depending on the study.

This risk is highest in the first 12 hours, with an incidence rate between 4 and 14% in the first 24 hours. It increases synchronously with the severity of the SAH according to the WFNS classification (9, 10, 12, 15,18).

## Intracranial hypertension and hydrocephalus

The irruption of blood into the subarachnoid spaces leads to an increase in intracranial pressure. This variation in pressure is sometimes such that the cerebral perfusion pressure becomes zero, causing loss of consciousness during intracranial hypertension (ICH) (12, 15, 19).

Prolonged ICH is the cause of significant sequelae related on the one hand to ongoing ischemia per hypertension, and on the other hand to cerebral edema following this ICH.

Intracranial pressure monitoring is necessary for the management of severe SAH via a PIC sensor or via DVE if there is associated hydrocephalus.

Hydrocephalus, particularly acute hydrocephalus, accounts for up to 25% of SAH cases. This is partly due to ventricular flooding that follows intracranial bleeding, which obstructs cerebrospinal fluid flow, causing ventriculomegaly and hydrocephalus (9, 12, 15, 19). This is a factor in poor prognosis.

As soon as it is identified, this complication justifies the implementation of a surgical treatment consisting of an External Ventricular Drainage (EVD) in order to limit the ICH.

## Arterial vasospasm

Arterial vasospasm is one of the most dreaded complications of SAH.

It corresponds to a spasm of the cerebral arterial lumen associated with microcirculation disorders. The peak incidence is between the 5th and 14th day, however vasospasm can appear as early as the 3rd day and up to 4 weeks after SAH. (15,20)

This vasospasm is responsible for the appearance of ischemic areas of the brain in sometimes very large proportions (30% of patients) (15,20).

Angiography remains the gold standard method for diagnosing vasospasm (20). The infusion scanner can also be used to make the diagnosis.

Treatment is based on the prevention of this vasospasm through the use of NIMODIPINE, which is fat-soluble and easily crosses the blood-brain barrier. **Administration of NIMODIPINE should be preferred orally** (including gastric tube).

***Administration of intravenous NIMODIPINE is subject to the risk of severe arterial hypotension and cerebral hypoperfusion, which should be taken into account in the benefit/risk balance.***

Treatment for proven vasospasm is based on localized intra-arterial injection of vasodilators (18,20).

## Other complications

- Neurologically: early (<24h) or late (>24h) seizures No prophylactic treatment has been established, nor is there a specific strategy (22).
- Cardiovascular: Endocardial lesions due to massive release of endogenous catecholamines (adrenaline and noradrenaline), arrhythmias (21).
- Pulmonary: pulmonary edema of neurogenic origin linked on the one hand to myocardial dysfunction and on the other hand to capillary hyperpermeability (21).
- Renal and metabolic: dysnatremia can be hyponatremia explained by 2 mechanisms, Cerebral Salt Wasting Syndrom or Inappropriate Secretion of Anti-Diuretic Hormone (SIADH). Only by carrying out additional examinations can a decision be made. The occurrence of hypernatremia is not excluded (3).

## Therapeutic management

Treatment for SAH involves securing the aneurysmal sac as quickly as possible. It is a therapeutic emergency requiring the patient to be taken care of in a specialized center and, if necessary, transferred if necessary in the event of an insufficient technical platform (10, 15, 23).

There are two main types of treatment, microsurgical or endovascular. The latter is currently the reference technique at national level (10, 16, 23, 24).


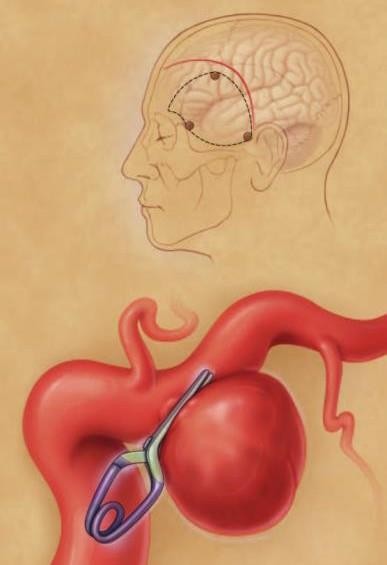

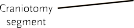

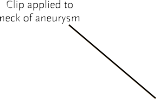

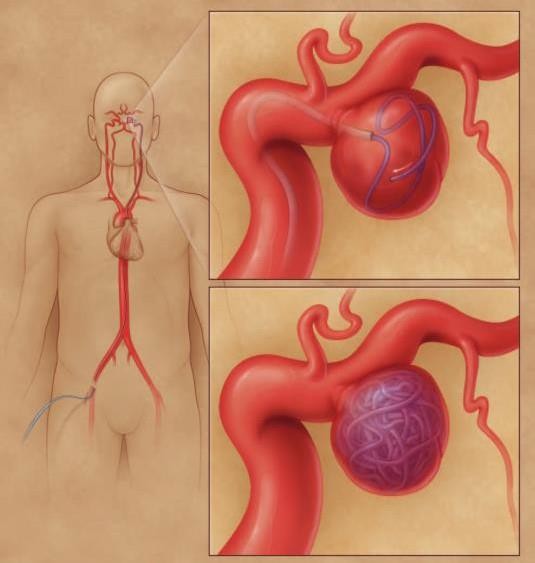


While waiting for the transfer to a specialized center, in this case the Rothschild Ophthalmological Foundation is the privileged partner of the University Hospital of Guadeloupe, appropriate care is recommended to prevent complications.

## Airway Control

The decision to intubate orotracheal with mechanical ventilation and sedation is based on the neurological examination and the severity of the coma and the constraints of transfer to an interventional radiology centre or EVD placement.

Induction of anesthesia should be done in rapid sequence with non-depressant hypnotics of the cardiovascular system (KETAMINE or ETOMIDATE)

The use of capnography monitoring is recommended as soon as possible.

## Prevention and treatment of vasospasm

Prevention of vasospasm is based on the use of NIMODIPINE **per os** 60 mg every 4 hours from the time of diagnosis until the patient is transferred to the referral centre (10).

***Administration of intravenous NIMODIPINE is subject to the risk of severe arterial hypotension and cerebral hypoperfusion, which should be taken into account in the benefit/risk balance.***

Maintenance of euvolemia and normal circulating blood volume is recommended.

Similarly, **early detection of vasospasm** using Transcranial Doppler is possible. Average speeds > 120 cm/s, an increase >50 cm/s, or a Lindegaard ratio >3 are highly suggestive (10, 25).

**For this reason, the performance of a transcranial Doppler at the entrance and regular monitoring is IMPERATIVE.**

The medical treatment of vasospasm is based on blood pressure optimization (MAP between 100 and 120 mmHg), control of secondary brain attacks of systemic origin.

## Management of ICH and hydrocephalus

On the one hand, the prevention of ICH is based on limiting the factors that can cause or aggravate it.

The presence of a frank ventricular flood should lead to the systematic and early initiation of a EVD before transfer (regardless of the Glasgow score).

In the absence of intracranial pressure monitoring and in a patient who is not neurologically eligible, daily transcranial Dopplers should be performed. PIs increased > 1.4 and diastolic velocities decreased < 20 cm/s are in favor of ICH and should be performed as a matter of urgency.

Osmotherapy has a place in the treatment of a flare-up of ICH **while waiting for a therapeutic procedure**. A prescription of mannitol 20% is recommended at the 250 mL renewable dosage, provided the patient is hemodynamically stable (euvolemic, RTA > 80mmHg). Hypertonic saline 3% has a comparable efficacy, but it has the advantage of not causing osmotic diuresis and associated hypovolemia and hypoperfusion.

| **MANNITOL 20%** | **Hypertonic Salty Serum** |
| --- | --- |
| Infusion 250mL in 15-20 min  (failing that, 500mL of Mannitol 10%) | 80 mL NaCl 0.9% + 20 mL NaCl 10% = 100 mL NaCl 3%  300mL infusion in 20 min |
| Caution: osmotic diuresis  Systematic compensation by  NaCl 0.9% | Caution: hypernatremia and hyperchloremia |
| Maximum effect in 10 to 15 minutes  Theoretical duration of 2 to 4 hours  Blood ionogram monitoring | |

Intraventricular fibrinolysis is contraindicated prior to surgical or endovascular securing of the aneurysm. Instead, a blocked EVE should be discussed before the patient leaves.

## Prevention of rebleeding

Blood pressure (BP) control should be performed. The goal is to ensure sufficient cerebral perfusion pressure by minimizing the risk of rebleeding as much as possible (this depends on ICP, pre-existing hypertension, vascular compliance, EVD). No specific objectives can be defined. A DTC assessment will allow the adjustment of the target values for each patient to be applied both in the ICU and during transport.

*Objectives : BP < 185 / 110 mmHg*

## General measures

Pain prevention and treatment remain essential during patient care.

The general measures concern the prevention of secondary cerebral attacks of systemic origin requiring:

- The proclive position + 30°
- Normothermia and the aggressive fight against hyperthermia
- Normoglycemia and the fight against hypoglycemia
- Normonatremia and the fight against hyponatremia
- Normocapnia and the fight against hypercapnia
- Combating hypotension with early introduction of catecholamines if necessary. The objective of MAP is to be adapted to the data of TCD or intraventricular or intracranial pressure. This should aim for a cerebral perfusion pressure *> 60 mmHg and < 80 mmHg*

As with MAP, no specific cerebral perfusion pressure objectives can be defined. The goal is to ensure sufficient cerebral perfusion pressure by minimizing the risk of rebleeding as much as possible (this depends on ICP, pre-existing hypertension, vascular compliance, EVD placement). A TCD assessment will allow the adjustment of the target values for each patient to be applied both in the ICU and during transport.

Vascular approach:

- Patients who are hemodynamically stable, amine-free, and easily perfusable in the periphery may not require a central catheter for transport.
- On the other hand, the monitoring of invasive pressure by the systematic placement of an arterial catheter seems essential for transport and for the first 24-48 hours at least.

| **TCD** | **Normal** | **ICH** | **Hyperemia** | **Vasospasm** |
| --- | --- | --- | --- | --- |
| **MCA** | Vsm = 100 +/- 20  Vm = 60 +/- 10  Vd = 40 +/- 10  PI = 1 +/- 0.2 | Vd low < 20 Vm low  Normal Vsm  IP high > 1.4 | Vd High Vm High IP Normal | Vm > 120  Vm + 50 cm/s on D+1  Lindegaard >3 |

# Administrative support

The use of a transatlantic transfer to a reference centre requires coordination between all the actors (intensive care specialists, neurosurgeons, emergency physicians, anesthetists).

In order to simplify this transfer, a number of steps must be taken:

## Contact details of the Reference Centre

Adolphe de Rothschild Ophthalmological Foundation:

In general, the Rothschild team is contacted directly by the neurosurgeons/ or the intensivists at the University Hospital of Guadeloupe.

## Organization of the transfer

1. **Information on the regulation of the Emergency Medical Service**

Inform the secretaries of the Intensive care unit in order to compile the administrative file and retrieve the patient's documents necessary for transport. In the event of transport at the weekend, the administrative file will be completed on Monday, but it is essential to collect the patient's identity papers necessary for the flight formalities from the family (identity documents, health insurance card and health insurance).

1. **Drafting of the various necessary documents**
   - - Letter to Medical Officer
     - 2 Transportation Vouchers:

UHG 🡺 Guadeloupe Airport (ambulance)

Orly Airport 🡺 Rothschild Foundation (ambulance)

- - - Request for prior agreement (UHG 🡺 Rothschild Foundation route)

1. **Medicaments**

Preparation of sedation syringes of PROPOFOL 10 mg/ml and SUFENTANIL 5 micrograms/ml +/- HYPNOVEL according to the request of the transporting physician.

Preparation of the number of NIMOTOP tablets required, i.e. 12 to cover a 24-hour duration.

Provide a syringe of ATRACRIUM 500mg/50 ml.

The total foreseeable duration of the transfer is approximately 16 hours in the absence of delay, so it is essential that the planned therapies cover a transfer duration of 24 hour

1. **References**
2. Zhang Y, Chapman A-, Plested M, Jackson D, Purroy F. The incidence, prevalence, and mortality of stroke in France, Germany, Italy, Spain, the UK, and the US: A literature review. Stroke Res Treat 2012.
3. Stroke Steering Committee, under the direction of E.Fery-Lemonnier. The prevention and management of strokes in France. 2009:1-161.
4. Audibert G, Puybasset L, Bruder N, Hans P, Berré J, Beydon L, et al. Severe subarachnoid haemorrhage: Natremia and renal function. Ann Fr Anesth Reanim 2005; 24(7):742-745.
5. Hankey GJ, Warlow CP. Treatment and secondary prevention of stroke: Evidence, costs, and effects on individuals and populations. Lancet 1999; 354(9188):1457-1463.
6. Dufour H, Bonafé A, Bruder N, Boulard G, Ravussin P, Lejeune J-, et al. Diagnosis in general hospital and immediate management of severe meningeal haemorrhages. Annales Françaises d'Anesthésie et de Réanimation, 2005, 7; 24(7):715-720.
7. Cowan Jr. JA, Dimick JB, Wainess RM, Upchurch Jr. GR, Thompson BG. Outcomes after cerebral aneurysm clip occlusion in the United States: The need for evidence-based hospital referral. J Neurosurg 2003; 99(6):947-952.
8. Cross III DT, Tirschwell DL, Clark MA, Tuden D, Derdeyn CP, Moran CJ, et al. Mortality rates after subarachnoid hemorrhage: Variations according to hospital case volume in 18 states. J Neurosurg 2003; 99(5):810-817.
9. Boulard G, Ravussin P, Proust F, Bonafé A, Audibert G, De Kersaint-Gilly A, et al. Organisation of care for patients suffering from subarachnoid haemorrhage. Ann Fr Anesth Reanim 2005; 24(7):721-722.
10. Proust F, Douvrin F, Gilles-Baray M, Levêque S. Treatment of aneurysmal meningeal hemorrhage. The Medical Press 2007 1; 36(1, part 2):150-157.
11. Connolly ES, Rabinstein AA, Carhuapoma JR, Derdeyn CP, Dion J, Higashida RT, et al. Guidelines for the management of aneurysmal subarachnoid hemorrhage: A guideline for healthcare professionals from the american heart association/american stroke association. Stroke 2012; 43(6):1711-1737.
12. Sztark F, Petitjean M, Thicoïpé M, Dabadie P. Meningeal haemorrhage due to aneurysmal rupture: initial management of the patient. Annales Françaises d'Anesthésie et de Réanimation, 1996; 15(3):322-327.
13. Losser M-, Payen D. Meningeal haemorrhage: management. Resuscitation 2007 10; 16(6):463-471.
14. Audibert G, Bousquet S, Charpentier C, Devaux Y, Mertes P-. Subarachnoid hemorrhage: epidemiology, predisposition, clinical presentation. Annales Françaises d'Anesthésie et de Réanimation 2007 11; 26(11):943-947.
15. Teasdale GM, Drake CG, Hunt W, Kassell N, Sano K, Pertuiset B, et al. A universal subarachnoid hemorrhage scale: report of a committee of the World Federation of Neurosurgical Societies. J Neurol Neurosurg Psychiatry 1988; 51(11):1457.
16. Albucher J. Update of the evaluation report on ruptured intracranial saccular aneurysms: endovascular occlusion versus exclusion by microsurgery. 2009;1:1-85.
17. Brisman JL, Song JK, Newell DW. Cerebral aneurysms. New Engl J Med 2006; 355(9):928-939.
18. Fisher CM, Kistler JP, Davis JM. Relation of cerebral vasospasm to subarachnoid hemorrhage visualized by computerized tomographic scanning. Neurosurgery 1980; 6(1):1-9.
19. Proust F, Dufour H, Lejeune J-, Bonafé A, De Kersaint-Gilly A, Puybasset L, et al. Severe subarachnoid haemorrhage: Treatment of rebleeding and of an intracerebral haematoma. Ann Fr Anesth Reanim 2005; 24(7):756-760.
20. Ter Minassian A, Proust F, Berré J, Hans P, Bonafé A, Puybasset L, et al. Severity criteria for subarachnoid haemorrhage: Intracranial hypertension, hydrocephalus. Ann Fr Anesth Reanim 2005; 24(7):723-728.
21. Berré J, Gabrillargues J, Audibert G, Hans P, Bonafé A, Boulard G, et al. Vasospasm after severe subarachnoid haemorrhage: Diagnosis and treatment. Ann Fr Anesth Reanim 2005; 24(7):761-774.
22. Hans P, Audibert G, Berré J, Bruder N, Ravussin P, Ter Minassian A, et al. Cardiovascular and pulmonary consequences of severe subarachnoid haemorrhage. Ann Fr Anesth Reanim 2005; 24(7):734-738.
23. Berré J, Hans P, Puybasset L, Beydon L, Audibert G, Bruder N, et al. Epilepsy in patients suffering from severe subarachnoid haemorrhage. Ann Fr Anesth Reanim 2005; 24(7):739-741.
24. Proust F, Bonafé A, Lejeune J-, De Kersaint-Gilly A, Gabrillargues J, Dufour H, et al. Severe subarachnoid haemorrhage: Aneurysm occlusion. Ann Fr Anesth Reanim 2005; 24(7):746-755.
25. Molyneux A, Kerr R, Stratton I, Sandercock P, Clarke M, Shrimpton J, et al. International Subarachnoid Aneurysm Trial (ISAT) of neurosurgical clipping versus endovascular coiling in 2143 patients with ruptured intracranial aneurysms: A randomised trial. Lancet 2002; 360(9342):1267-1274.
26. Piednoir P, Geeraerts T, Leblanc P-, Tazarourte K, Duranteau J, Vigué B. Early diagnostic for vasospasm after aneurysmal subarachnoid haemorrhage. Ann Fr Anesth Reanim 2007; 26(11):965-972.
27. Fauvage, Bertrand, et al. "Management of subarachnoid hemorrhage (SAH) by aneurysm rupture
